# Exploring Links between Polychronicity and Job Performance from the Person–Environment Fit Perspective—The Mediating Role of Well-Being

**DOI:** 10.3390/ijerph17103711

**Published:** 2020-05-25

**Authors:** Tung-Ju Wu, Jia-Ying Gao, Lian-Yi Wang, Kuo-Shu Yuan

**Affiliations:** 1 School of Management, Harbin Institute of Technology (HIT), No.92 West Dazhi Street, Nan Gang District, Harbin 150001, China; tjwu@hit.edu.cn (T.-J.W.); JiayingGao@outlook.com (J.-Y.G.); lynnwly1@outlook.com (L.-Y.W.); 2 Business School, Huaqiao University, No.269, Chenghuabei Road, Quanzhou 362021, China

**Keywords:** polychronicity fit, job performance, well-being, person–environment fit

## Abstract

Polychronicity refers to the preference of some individuals to structure their time in order to deal with multiple tasks simultaneously in a short period of time. Past research regarding the correlation between individual polychronicity and performance presented distinct arguments. Although most studies supported a positive correlation with performance, empirical findings showed inconsistent results, indicating the presence of other influencing factors. According to the person–environment fit theory and self-determination theory, the effect of polychronicity on job performance was verified and the mediation effect of well-being was tested in this study. Dual-mode questionnaires were collected from 532 subordinators and their direct supervisors in 98 chain restaurants and hierarchical regression analysis was performed to test the research hypotheses. The results showed that polychronicity positively affected well-being, that is, well-being was a full mediator between polychronicity and job performance. This study provides valuable insight for managers to understand employee polychronicity and, in turn, improve their well-being, which could help improve job performance.

## 1. Introduction

Previous research indicated that individual preference regarding time management is affected by organizational culture, personal experience, and the work environment, further influencing work attitudes, behaviors, and performance [1,2,3,4]. The modern workplace has gradually approached a multitasking philosophy [4,5], requiring employees to deal with several tasks simultaneously. Research revealed that the current organizational culture and individual work processing encouraged multitasking [2,6,7,8]. The idea of the preference for dealing with more than two tasks in a short period of time was termed “polychronicity”. Poposki and Oswald [9] further indicated that in addition to individual “preference” for work modes, polychronicity was the most efficient way to complete tasks. Therefore, the time management of employees in the workplace is particularly important. However, multitasking results in terms of individual performance did not present a consistent conclusion, possibly because the studies ignored the importance of individuals’ use of time, thereby not necessarily proving that multitasking was directly related to performance, and the person–environment fit was not taken into account. According to König and Waller [6], environment fit was applied in this study to replace single trait theory in order to clarify the relationship with performance. Although prior studies discussed the concept of person–job fit in regard to multitasking research, most include single-adaptive aspects, such as person–job fit or person–team fit [2,5,6,9], whereas “fit” is actually a multidimensional construct. In addition, the issue of which factors affect individual job performance regarding the process of polychronicity in the person–environment relationship was not discussed and clarified. Accordingly, the inconsistent results of polychronicity and performance induced the motivation of this study.

Polychronicity, a personal preference for a specific work mode, is directly correlated with job performance; however, directly discussing the relationship with performance encompasses trait theory [9]. As a two-way concept, “fit” takes individual traits and environmental characteristics simultaneously and considers the interaction between the two concepts to present better compatibility regarding the explanation of personal behavior [6]. For this reason, the idea of person–environment fit for polychronicity was proposed in this study. Furthermore, we focused on task performance measurement, which is directly correlated with individuals’ use of time. Finally, we attempted to clarify the relationship between polychronicity fit and performance and contributes to filling in this theoretical gap. Furthermore, among past studies on the relationship between polychronicity fit and performance, Kirchberg et al. [10] discovered that more individual and team polychronicity led to individuals being more willing to contribute efforts, stay in the organization, and agree with organizational objectives (i.e., higher organizational commitment), resulting in higher performance evaluations from supervisors and members [4]. In their research on need–supply fit in the person–job relationship, Hecht and Allen [11] discovered that higher levels of polychronicity in individuals was linked with higher levels of well-being and self-efficacy.

Past research proved that person–environment polychronicity may enhance individual work attitudes (e.g., organizational commitment and job satisfaction) [12]; however, the method in which polychronicity affected individual job performance was not further discussed. Past studies confirmed that multitasking may improve individuals’ organizational commitment and job satisfaction, but these variables could not directly explain how polychronicity affects individual performance. Although polychronicity may enhance organizational commitment, past research works pointed out that the impact of organizational commitment on job performance could be mixed, weak, or even negative [2,3,9,10]. Therefore, certain mediations between polychronicity and job performance in this were considered in this study; in other words, after individual fit with a work situation, this relationship would affect the individual’s mental state to further influence job performance. Previous studies indicated that organizational members’ well-being might be the mediator of this relationship; besides, most literature proved and agreed that well-being is an important antecedent to job performance [8,13,14,15]. Di Blasio et al. [16] explained that achievement of the employee–work role relationship satisfied employees’ psychological meaningfulness and further affected well-being, while also indicating that self-determination motivation satisfies distinct psychological needs through perceived environment fit, in accordance with self-determination theory [17].

Finally, it was verified in various studies that the person–environment fit presented multidimensional ideas [18,19]. From the literature review, person–organization (P–O), person–job (P–J), and person–supervisor (P–S) fits showed close relationships with employee work attitudes and job performance, significantly enhancing employee well-being [16]. This study aimed to clarify the correlation between polychronicity and performance using the “fit” approach in employees and supervisors of chain restaurants while using psychological condition theory to discuss how person–environment polychronicity fit affects individual job performance with regard to well-being.

### 1.1. Polychronicity and Person–Environment Fit Theory

Polychronicity is the preference for simultaneously executing several tasks in a short period of time. Successive research mostly extended this point of view and regarded polychronicity as the preference to simultaneously deal with several affairs [10]. Polychronicity contains two elements of time and multitasking, with time describing the idea of “at a time” or “in a short time”. Past research on simultaneous multitasking often defined the time concept of polychronicity as “at a time” or “simultaneously”; however, König and Waller [6] considered that these terms did not refer to a physical snapshot of time but rather a period of time. König and Waller [6] defined a short time as being within an hour. In this study, “multitasking” focused on work-related tasks, while daily behaviors not related to work tasks were not covered. In summary, polychronicity is defined as an individual preference for dealing with several tasks simultaneously in a short period of time.

“Fit” refers to the mutual function between an individual and their environment. Fit is a two-way function, with person–environment fit referring to the supply–need balance between an individual and their environment [19]; individual–environment interaction factors should be taken into account to effectively explain an individual’s behavior in their environment. Through this mutual function, analyses and discussions between the individual and their environment could help to clarify the real relationship between the two factors. The discussion of person–environment fit could avoid errors caused by separate consideration of the individual or the environment [18].

Some researchers considered that different types of fit played various roles in the person–environment relationship to show distinct points [18,19]. For instance, person–job fit refers to the compatibility between an individual and specific work [11]. Person–organization fit is the fit of an individual with organizational values, goals, and missions [20]. Person–team fit discusses the abilities of an individual and an organizational team, as well as the comparison of interpersonal relationships [20]. Person–job fit appears to have the most significant effect on an individual’s job selection, as individuals tend to initially select a job matching their abilities and personality traits [18]. Person–other fit discusses the dual relationship between an individual and other member of the work environment, including relationships among colleagues, between candidates and recruiters, and between mentors and students. Supervisor–subordinate fit, i.e., person–supervisor fit, was broadly discussed [21]. In the workplace, an individual does not simply engage with work, but must interact with various dimensions of the environment. In modern organizations, person–organization (P–O), person–job (P–J), and person–supervisor (P–S) relationships are comparatively closer [12,18,22]. Therefore, we regarded these three dimensions in terms of polychronicity to discuss the relationship with well-being in this study.

### 1.2. Polychronicity Fit and Well-Being

Job fit refers to the fit between a person and their job [19] including two dimensions: (1) demand–ability fit, i.e., individual knowledge, skill, and abilities (KSAs) matching work requirements, and (2) need–supply fit, i.e., work characteristics conforming to individual desire and needs, covering objectives, psychological needs, interests, rewards, and values.

In organizational behavior research, well-being was proposed to discuss employees’ opinions about and attitudes toward work. Well-being referred to an individual engaging in, being satisfying with, and enjoying the work [8,23]. Obrenovic et al. [24] considered that well-being presented sustainability, proactivity, and strong motivation to work. According to the psychological condition theory, the antecedent of well-being refers to employees being motivated and exhibiting well-being when psychological conditions, such as psychological meaningfulness, psychological safety, and psychological availability, were satisfied [8,25]. Moreover, self-determination theory describes how individual motivation is affected by the social environment through distinct satisfaction of psychological needs, with the perceived environment further forming motivation due to self-determination. Since person–job fit is regarded as the fit between an individual and their job, this study considered that an employee would achieve polychronicity when the individual’s ability to structure time matched their ability to multitask. In other words, when an employee’s time-structuring tendency matched with the task requirements or attributes, the employee would be more likely to perform better, thereby satisfying their psychological meaningfulness. According to psychological condition theory, an employee would realize work environment factors (e.g., interpersonal, group, position, and organization) through satisfaction with the three psychological conditions (meaningfulness, safety, and effectiveness), thereby improving their well-being. It was therefore proposed in this study that H1a: person–job polychronicity fit would show a significant positive relationship with well-being.

Chatman [26] proposed person–organization fit (P–O fit) and considered that selection and socialization between individuals and organizations should be taken into account when studying and predicting employee behaviors. Meyer et al. [12] pointed out that person–organization fit was a cultural fit based on consistent values between individuals and organizations. In this case, when an employee’s preference for time-structuring matched with the time-use preferences of the organization or their peers, an individual would perceive better organizational climate, greater security, greater agreeance with the organization, and more meaningful work [20]. Xu et al. [25] revealed that employees would generate well-being motivation when the psychological conditions were satisfied. When an employee appeared to have consistent values with the organization, the employee would observe positive job performance and show positive work attitudes. In this case, person–organization polychronicity referred to an organization presenting the organizational culture of dealing with several tasks in a short period of time. When the individual’s value of time matched the organization’s, the employee would show positive work attitudes, concentrate better on the work, and be more willing to devote themselves to the work, thereby finding delight in the work. Therefore, we proposed in this study that H1b: person–organization polychronicity fit would reveal a significant positive relationship with well-being.

Person–supervisor fit refers to the fit between an individual and the supervisor [21], where the factors within the fit include supervisor leadership style and individual traits, values, and preferences. Research indicated that supervisors showing similar preferences, personality traits, living backgrounds (including age, gender, race, education, seniority), and work modes with their subordinates had higher interpersonal attraction for both parties, resulting in greater interaction frequency and higher subordinate job satisfaction [20]. Carmona-Halty et al. [27] discovered that operators with consistent work values with the supervisor had higher satisfaction and organizational commitment; therefore, they were more willing to commit to and concentrate on the organization. Furthermore, the interaction of supervisor and subordinate time-structuring preferences affected the subordinate’s opinions regarding the job. When a supervisor presented consistent time use-value with their subordinates, the interaction relationship was better, and the subordinates could fully present themselves [16]. Based on this, a subordinate did not worry about negative evaluation from their supervisor affecting promotions, rewards, or future career development. Accordingly, the perceived secure work environment allowed them to concentrate on their work [28]. It was therefore proposed in this study that H1c: person–supervisor polychronicity fit would show a significant positive relationship with well-being.

### 1.3. Well-Being and Job Performance

Job performance refers to an employee’s performance at work. Miao and Cao [29] divided job performance into three factors: efficiency, productivity, and effectiveness. LePine et al. [30] defined job performance as all behaviors related to organizational objectives, which could be measured according to individual contributions to organizational objectives and divided into task performance and contextual performance. Task performance was defined as being familiar with relevant work skills to complete tasks, mainly referring to the degree of employee achievement in regard to the work objectives expected by the organization. The contextual performance was not related to major task performance, but indirectly assisted in the completion of tasks, including voluntarily executing informal tasks, helping others and cooperating with others, and following organizational rules and procedures. Therefore, in this study, sustainable job performance was defined as the degree of an employee achieving the work objectives expected by the organization, focusing on the presentation of task performance. Employees with high well-being presented positive attitudes, while those with low well-being showed negative attitudes. Factors of well-being included the work being mentally stimulating, fair rewards, and a supportive work environment and colleagues. Past research regarded job satisfaction as the way employees perceive the job, as well as individual attitudes toward various work evaluations. Since employees with positive well-being are loyal to their organizations, show a strong sense of honor and work autonomy, and unconditionally invest more time and energy, we proposed that H2: well-being would show a significant positive relationship with job performance.

### 1.4. The Mediation Role of Well-Being

According to the self-determination theory, individual intrinsic motivation is affected by the environment; in other words, an individual achieves distinct satisfaction of psychological needs through a perceived environment to form motivation via self-determination, thereby affecting behavior and performance [15,16]. Psychological condition theory also proposes three psychological conditions at work to induce employee motivation and well-being, namely psychological meaningfulness, psychological security, and psychological availability. Person–job fit was regarded as the fit between a person and their job in which the employee’s time-structuring mode matched the multitasking characteristics to achieve polychronicity [31] and positively promote well-being [16]. According to the above theoretical bases, well-being refers to the individual motivational construct at work and describes an employee’s positive and practicable ambition and the emotional motivation resulting from the work [32].

Research on polychronicity also indicated that person–environment fit was a primary predictive variable for some psychological motivation variables, such as organizational commitment, job satisfaction, and well-being [11,19,33,34]. Nerstad et al. [28] discovered that when an individual, supervisors, and team members showed higher polychronicity, the individual was more willing to make an effort, present higher vitality and concentration, agree with organizational objectives, and acquire higher performance evaluations from supervisors and members, even though the surveyed organization was not a multitasking organization. For this reason, when the time-structuring characteristics of an employee fitted the work environment (including work, partners, and supervisors), a person–environment fit in terms of polychronicity was simultaneously achieved. It was therefore proposed in this study that H3: employees’ well-being would mediate the relationship between person–environment polychronicity and job performance; more specifically, that H3a: employees’ well-being would mediate the relationship between person–job fit and job performance, H3b: employees’ well-being would mediate the relationship between person–organization fit and job performance, and H3c: employees’ well-being would mediate the relationship between person–supervisor fit and job performance.

**Figure 1:**
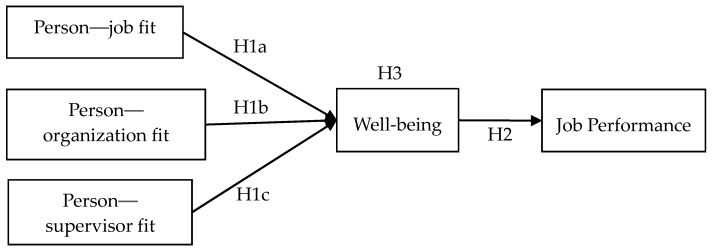
The framework of person–environment polychronicity, well-being, and job performance.

Figure 1 presents a diagram of our research model.

## 2. Materials and Methods

### 2.1. Participants and Procedures

A restaurant chain in Fujian Province, China, was selected as the research population, with the workers sampled in this study. Questionnaires were distributed to the employees of chain restaurants in Fuzhou, Quanzhou, and Xiamen, including 98 supervisors and 700 subordinates. Before the questionnaire distribution, the contact persons in the units were contacted and the questionnaire content, way of response, and relevant remarks were explained to the participants. The questionnaire was filled out from 1 May 2019 to 3 August 2019. A total of 98 supervisor questionnaires and 577 subordinate questionnaires were recovered. After excluding invalid samples, 98 supervisor and 532 subordinate questionnaires were used; the overall questionnaire recovery was 76%.

The characteristics of the study participants are shown in Table 1. Two types of dual-mode questionnaire were filled out by supervisors and subordinates. The supervisor questionnaire contained the Polychronic Attitude Index (PAI) [35] and job performance, while the subordinate questionnaire covered Polychronic Attitude Index (PAI), Inventory of Polychronic Values (IPV) [36], polychronicity fit, and well-being. A 6-point Likert scale (1 = “never” to 6 = “always”) was utilized for measurement in this study. Higher scores represented greater agreement with the content of the factor.

### 2.2. Measures

*Person–organization fit* is the consistency between individual preference and organizational values for dealing with several tasks in a short period of time. The absolute difference between the mean of the Polychronic Attitude Index (PAI) proposed by Bluedorn et al. [35] and the mean of the Inventory of Polychronic Values (IPV) proposed Bluedorn et al. [36] was used for this measurement. Four items of the PAI were presented, including, “I feel that dealing with multiple tasks in a short time could smoothly precede unit tasks” and “I feel that dealing with several affairs in s short time is reasonable”. Items for the IPV included 10 items, e.g., “Our unit tends to deal with/participate in several tasks and activities simultaneously in a short time” and “In comparison with completing one thing in a day, our unit tends to execute several affairs at a time in a day”. The Cronbach’s α for PAI was 0.92 and the Cronbach’s α for IPV was 0.91.

*Person–job fit* refers to the consistency of a person and their work [19]. The consistency between an individual’s polychronic attitude and actual polychronicity was used for measurement in this study. This measurement was adapted from König et al. [5] and describes the absolute value of the multitasking scale plus the total average score minus the average score of the individuals’ PAI. Items for the polychronicity scale included five items, e.g., “I often need to deal with several affairs simultaneously in my job” and “In my job, I am often asked to complete several tasks in a short time”. The Cronbach’s α for this scale was 0.87.

*Person–supervisor fit* refers to the fit between a person and their supervisor. The consistency between a supervisor’s and the subordinate’s simultaneous multitasking value was used in this study to measure polychronicity [5]. The variable was measured using the absolute difference between the mean of individual PAI and the mean of the supervisor’s PAI. The Cronbach’s α of the supervisors for this scale was 0.86 and the Cronbach’s α of the subordinates for this scale was 0.88.

*Well-being,* according to Zheng et al.’s (2015) [37] scale, was used to measure target employees’ well-being in their work environments. Specifically, there were six items in this scale, for example, “I am satisfied with my work responsibilities”, “in general, I feel fairly satisfied with my present job”, “I find real enjoyment in my work”, “I can always find ways to enrich my work”, “work is a meaningful experience for me”, and “I feel basically satisfied with my work achievements in my current job”. The Cronbach’s α for this scale was 0.92.

*Job Performance* refers to observing or measuring the proficiency of the employee engaging in activities under individual control and with contribution to their organization. This scale was revised from the scale proposed by LePine et al. [30], including four items. The Cronbach’s α for this scale was 0.91.

## 3. Results

The hypothesized model using confirmatory factor analysis exhibited good fit to the data, as shown in Table 2 (χ^2^(575) = 1414.52, *p* < 0.001; RMSEA = 0.06, SRMR = 0.05, TLI = 0.91, CFI = 0.96). According to the χ^2^ test, the five-factor theoretical model showed a more favorable factor structure than the four-factor model (Δχ^2^(22) = 36.19, *p* < 0.001), the three-factor model (Δχ^2^(25) = 217.48, *p* < 0.001), the two-factor model (Δχ^2^(27) = 355.36, *p* < 0.001), or the one-factor model (Δχ^2^(28) = 530.81, *p* < 0.001). Table 3 presents the correlations, means, and standard deviations of the study variables.

**Figure 2:**
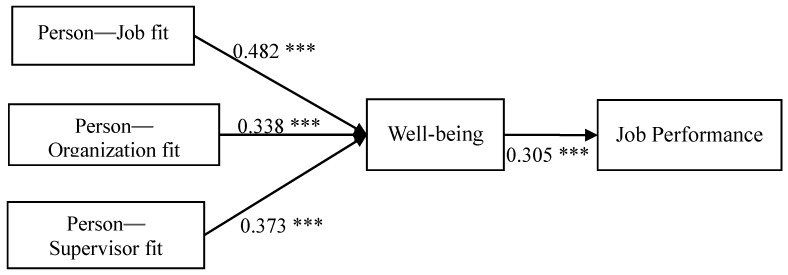
Path analysis results, *** *p* < 0.001.

STATISTICA 12.0 and AMOS 23.0 were used for the analyses in this study. Regarding the hypothesis test, H1a–c mainly tested the effects of person–environment polychronicity fit on well-being. The independent variable was calculated using the absolute difference between the individual and the environment and corrected through the square root of reliability, which was substituted into the path coefficient. The results showed significantly positive effects of person–job fit on well-being (γ = 0.482, t = 7.973), thereby supporting H1a. Person–organization fit also revealed significant positive effects on well-being (γ = 0.338, t = 6.852), supporting H1b, and person–supervisor fit presented significant positive effects on well-being (γ = 0.373, t = 7.317), supporting H1c. Well-being also appeared to have significantly positive effects on job performance (γ = 0.305, t = 6.524), supporting H2. The structural model analysis results of the main effects and correlation coefficients are shown in Figure 2.

Well-being was regarded as the mediator between polychronicity fit and job performance in the theoretical model. The Sobel test was utilized in this study, whereby path analysis was conducted in the contexts of person–job fit, person–organization fit, and person–supervisor fit through well-being to job performance; the test standard appeared when Sobel Z was higher than 1.96, revealing a significant effect, with α = 0.05, i.e., a significant mediating effect. These test results are shown in Table 4. Bootstrap was also applied in this study to test the correlation coefficients 5000 times. Within the 95% interval, the mediating effect of well-being on polychronicity fit and job performance did not contain 0, revealing a notable mediation effect. The overall results are shown in Table 5.

## 4. Discussion

Various previous studies of polychronicity focused more on the demographic variables of employees. However, this study focused on the issue of polychronicity regarding employees’ use of time. According to the person–environment fit theory and the self-determination theory, the multitasking work pattern is becoming increasingly utilized, therefore, we adapted polychronicity fit as the main variable to discuss its effect on job performance and the mediating effect on well-being. This encompassed a research gap not previously mentioned in other studies. Therefore, this study expanded the research scope of the satisfaction of psychological needs in terms of the relationship between person–environment polychronicity and individual job performance. This study aimed to test the effects of polychronicity fit (person–job, person–organization, and person–supervisor on individual job performance through well-being in a work environment). The research results revealed significantly positive effects of person–environment fit on polychronicity (including person–job, person–organization, and person–supervisor fit) on well-being in a work environment, demonstrating that an individual would show greater well-being and motivation under high fit. Second, individual well-being also presented positive and notable effects on sustainable job performance, revealing that individual job performance was affirmed and approved by the supervisor when the person was willing to contribute to the organization and provide individual value. Finally, the mediation effect of well-being on the relationship between person–environment fit on polychronicity and job performance also achieved significance, revealing that a better fit of polychronicity (containing person–job, person–organization, and person–supervisor fit) affected job performance via improved well-being in a work environment. Promotion of individual job performance by enhancing person–job fit on polychronicity was more significant, that is, by offering tasks with greater multitasking capabilities for polychronic employees with polychronicity, with employees concentrating on single tasks offered tasks with high simultaneous time use in order to increase employee well-being and motivation to generate better work effects.

### 4.1. Theoretical Contributions

A lot of research regarding organizations proposed combining the viewpoint of “fit”, expecting future research with extension and situational interaction [6,20]. This study, as one of few studies on the mediation of fit from the aspect of psychological need satisfaction, proposed a new angle for the relationship between person–environment fit and job performance. Past research on fit mainly discussed the relationship between distinct person–environment fit (e.g., person–organization, person–job, and person–supervisor) and job performance. This study, however, focused on polychronic employees’ preferences for or values toward time-structuring in the workplace, verifying and responding to the hypothesis of individual preference for time use would be affected by the organization, supervisors, and work environment characteristics to further affect work attitudes, behaviors, and performances proposed in research on polychronicity [19,20]. We further responded to the discussion of the person–situation interaction and brought the simultaneous multitasking of individuals in trait theory into multi-oriented person-environment fit theory for in-depth analysis. Empirical research could be conducted to investigate individual and organizational time use in regard to the impact of individual time management on job performance. Our results not only showed that this would help individuals complete tasks and improve job performance with consistent values of the individual and the workplace, they also clarified why employees who prefer to deal with multiple tasks in a short time have lower job performance when the values are inconsistent. Based on self-determination theory and psychological condition theory, we established the rationality of the mediation relationship between person–environment polychronicity fit and job performance from the aspect of psychological need satisfaction. The effect of fit theory in the workplace was extended, and we responded to the argument of self-determination theory that individuals with satisfied psychological needs would present higher autonomy during relevant activities [13,23,30]. Previous research on the job demand resource model discovered that the offer of work resources, such as employee welfare, education and training, work autonomy, and challenge, significantly promoted well-being [28,30]. In this study, the research results revealed that polychronicity fit significantly predicted an employee’s well-being, thereby filling in the gap of past research using work resource indicators for predicting employee well-being.

### 4.2. Implications for Practice

In management practice, these research results could provide substantial suggestions for the recruitment of employees in the service industry. First, the recruitment of employees in the service industry should stress the measurement of “position fit” to understand the fit of personnel to job attributes in order to achieve consistency between employees and organizations in terms of use and of time. In this case, employee well-being could be enhanced to avoid inconsistent time-structuring tendencies resulting in lack of enthusiasm, poor concentration, and low performance. Measurements related to fit in regard to polychronicity, such as “position fit” or “adaptability”, should therefore be added to recruitment processes, in addition to existing tests, in order to select practitioners suitable for the service industry. Second, in order to achieve good performance, organization managers should understand subordinates’ preferences and values in order to select suitable subordinates for the work. In this case, subordinates would engage more with the work to further achieve good job performance. Finally, job, organization and supervisor polychronicity were compared in this study, with the effect of person–job polychronicity fit showed to be greater than that of organization and supervisor fit. For managers, in order to enhance subordinate well-being, subordinates should be fit to engaged work to enhance well-being. For this reason, when assigning work, managers should carefully consider the fit between work attributes and subordinates’ abilities and polychronicity should be executed.

### 4.3. Limitations and Future Research

The data collected in this study were single time-point data in a cross-sectional study. As such, a cause–effect relationship could not be directly proven among the variables. Nevertheless, the variable model relationship followed self-determination theory and matched cognition–psychological motivation (mediation) and behavior evaluation (performance), with the research results presenting reference values on the cause–effect inference. Moreover, samples from other cities in Fujian Province were not acquired; the characteristics of the service industry in different areas may be distinct. However, stratified sampling was utilized in this study. Although the samples might be applicable for employees in the service industry, they cannot be extrapolated to various industries in society. After all, profit-seeking enterprises are different from non-profit-seeking ones. Extending the research to different industries would be another issue to address in successive research.

## 5. Conclusions

Person–environment fit theory and self-determination theory were utilized in this study to discuss the relationships between polychronicity fit, well-being, and job performance in chain restaurant employees in China. Polychronicity fit (person–job, person–organization, and person–supervisor) was found to affect individual job performance through well-being in the work environment, that is, tasks with higher multitasking should be offered to employees with polychronicity, while employees concentrating on single tasks should be offered tasks with high simultaneous simplex time use so that employees overall are more motivated and achieve better well-being in order to generate better job performance. Finally, the results revealed that polychronicity fit significantly predicted an employee well-being, thereby filling in the gap left by past research using work resource indicators to predict employee well-being. Supervisors should pay more attention to their employees’ well-being.

## Figures and Tables

**Table 1:** Participant characteristics.

Variable	Supervisor/Subordinate	Category	*n*	%
Gender	Supervisor	Male	75	77%
		Female	23	23%
	Subordinate	Male	172	32%
		Female	360	68%
Age	Supervisor	31.2 (SD = 4.22)		
	Subordinate	23.6 (SD = 6.84)		
Education	Supervisor	Below university	53	54%
		University or above	45	46%
	Subordinate	Below university	415	77%
		University or above	125	23%
Job Tenure	Supervisor	9.7 (SD = 5.73)		
	Subordinate	2.4 (SD = 3.32)		

**Table 2:** Measurement model test.

Models	χ^2^	*df*	Δχ^2^	RMSEA	SRMR	TLI	CFI
1. Baseline model (including P–J fit, P–O fit, P–S fit, WB, JB)	1414.52	575	-	0.06	0.05	0.91	0.96
2. Four-factor model (combining P–J fit and P–J fit into one factor)	1450.71	597	36.19 ***	0.09	0.09	0.88	0.90
3. Three-factor model (combining P–J fit, P–O fit, and P–S fit into one factor)	1632	600	217.48 ***	0.13	0.14	0.84	0.83
4. Two-factor model (combining P–J fit, P–O fit, and P–S fit into one factor and WB and JP into one factor)	1769.88	602	355.36 ***	0.19	0.21	0.81	0.78
5. One-factor model (combining all items into one factor)	1945.33	603	530.81 ***	0.25	0.29	0.72	0.74

*n* = 532. * *p* < 0.05, ** *p* < 0.01, *** *p* < 0.001. P–J fit = person–job fit; P–O fit = person–organization fit; P–S fit = person–supervisor fit. df = degree-of-freedom.

**Table 3:** Mean scores, standard deviations, and correlations among study variables.

Variable	μ	SD	1	2	3	4	5	6	7
Subordinator (*n* = 532)									
1 Age	23.6	6.84	−						
2 Gender	0.32	0.46	0.05	-					
3 Job tenure	2.4	3.32	0.18 *	0.33 **					
4 P–J fit	3.41	1.68	0.22 *	−0.14	0.31 **				
5 P–O fit	3.15	1.73	0.24 *	−0.15	0.34 ***	0.28 **			
6 P–S fit	3.08	1.54	0.21 *	0.18 *	0.27 **	0.22 *	0.24 *		
7 Well-being	3.88	1.85	0.27 **	−0.23 *	0.25 **	0.31 **	0.29 **	0.35 ***	
8 Job performance (from supervisor)	3.29	1.67	0.25 **	−0.19 *	0.33 **	0.28 **	0.35 ***	0.29 **	0.35 ***
**Supervisor (*n* = 98)**									
Age	31.2	4.22	−						
Gender	0.77	0.68	0.11						
Job Tenure	9.7	5.73	0.24 *	0.14					
P–S fit	3.47	1.32	0.21 *	0.25 **	0.27 **				

* *p* < 0.05, ** *p* < 0.01, *** *p* < 0.001; μ refers to mean; SD refers to standard deviation. P–J fit refers to person–job fit; P–O fit refers to person–organization fit; P–S fit refers to person–supervisor fit. Gender:1 = male and 0 = female.

**Table 4:** Mediating effect of well-being by the Sobel test.

Path	P	Z
P–J fit--> well-being --> job performance	0.001	4.63 ***
P–O fit--> well-being --> job performance	0.001	3.75 ***
P–S fit--> well-being --> job performance	0.001	4.18 ***

*n* = 532. * *p* < 0.05, ** *p* < 0.01, *** *p* < 0.001. P–J fit = person–job fit; P–O fit = person–organization fit; P–S fit = person–supervisor fit.

**Table 5:** Mediating effect of well-being by Bootstrap.

Path	CI 2.5%	CI 97.5%
P–J fit--> job performance	0.042	0.343
P–O fit--> job performance	0.003	0.228
P–S fit--> job performance	0.027	0.271
